# Polyphenol Concentrate from Kazakhstan Cabernet Sauvignon Collection of Grapes

**DOI:** 10.5195/cajgh.2014.174

**Published:** 2014-12-12

**Authors:** Zarina Shulgau, Vladislav Tritek, Alexander Gulyaev, Gulsim Adilgozhina, Talgat Nurgozhin

**Affiliations:** Center for Life Sciences, Nazarbayev University, Astana, Kazakhstan

**Keywords:** gerontology, polyphenol, longevity, antioxidants

## Abstract

**Introduction:**

Nowadays, most of the research in the field of gerontology is focused on the effects of the grape polyphenols. In particular, resveratrol has been shown to increase life expectancy of various living organisms, including mammals. Resveratrol also plays an important role in cancer prevention and decreases the risk of developing cardiovascular disease. In our research, we proposed the development of the therapeutic product from Cabernet Sauvignon grapes that would exhibit the beneficial properties of polyphenols. Standard operating procedures were developed in our laboratories to collect alcohol free concentrate of polyphenols from the Kazakhstan Cabernet Sauvignon collection of grapes. The purpose of the study was to investigate the composition, biological safety, and potential therapeutic effects of the polyphenol concentrate.

**Methods:**

The total polyphenol amount was determined using the Enology Analyzer Y15 (BioSystems, Spain). HPLC analysis of the polyphenol composition was performed using Agilent 1290 chromatograph. The polyphenol concentrate was analyzed for the microbiological purity and the presence of the toxic elements. The cytoprotective effect of the polyphenol concentrate was studied in experimental models of diabetes, toxic hepatitis, doxorubicin cardiomyopathy, and acute radiation sickness.

**Results:**

The total polyphenol amount in one sample was 12,819 mg/l. Polyphenol composition analysis showed presence of the following polyphenols: catechin, epicatechin, gallic acid, quercetin, miricetin, 3-glucosylkaempferol, epicatechin gallate, 3-(3,4-Dihydroxyphenyl)-2-propenoic acid, catechin gallate, pitseid, kaempferol, n-hydroxy-cinnamic acid, resveratrol and chlorogenic acid. The concentrate was proven to be biologically safe and acceptable for use as a dietary supplement. The polyphenol concentrate demonstrated high antioxidant activity against ABTS and DPPH radicals in vitro. It also showed the following impacts on the various experimental models in vivo: reduction of sugar levels in diabetes; regeneration of the structure and function of the heart tissue in cardiomyopathy; regeneration of the nephron structure and function in nephropathy; regeneration of liver in toxic hepatitis; recovery of the antioxidant status in oxidative stress; and recovery of the hematopoiesis in acute radiation sickness.

**Conclusion:**

The polyphenol concentrate from Kazakhstan Cabernet Sauvignon collection of grapes was proved to be biologically safe and acceptable for use as a dietary supplement. The concentrate showed high antioxidant, antiradiation activity, and regenerative effect in diabetes, cardiomyopathy, nephropathy, and hepatitis in the corresponding organs.

